# Antihypertensive Properties of Plant-Based Prebiotics

**DOI:** 10.3390/ijms10083517

**Published:** 2009-08-10

**Authors:** Siok-Koon Yeo, Lay-Gaik Ooi, Ting-Jin Lim, Min-Tze Liong

**Affiliations:** School of Industrial Technology, Universiti Sains Malaysia, 11800 Penang, Malaysia; E-Mails: siokkoon85@yahoo.com (S.Y.); ooilaygaik@yahoo.com (L.O.); ericlimtingjin@hotmail.com (T.L.)

**Keywords:** prebiotics, mechanism, evidence, controversies, inulin, fos, fiber, gum

## Abstract

Hypertension is one of the major risk factors for cardiovascular disease. Although various drugs for its treatment have been synthesized, the occurring side effects have generated the need for natural interventions for the treatment and prevention of hypertension. Dietary intervention such as the administration of prebiotics has been seen as a highly acceptable approach. Prebiotics are indigestible food ingredients that bypass digestion and reach the lower gut as substrates for indigenous microflora. Most of the prebiotics used as food adjuncts, such as inulin, fructooligosaccharides, dietary fiber and gums, are derived from plants. Experimental evidence from recent studies has suggested that prebiotics are capable of reducing and preventing hypertension. This paper will discuss some of the mechanisms involved, the evidence generated from both *in-vitro* experiments and *in-vivo* trials and some controversial findings that are raised.

## 1. Introduction

Hypertension is defined as an elevation in blood pressure, where a blood pressure exceeding 140/90 mm Hg is considered as elevated, and a lower threshold of 130/80 mm Hg was applied for individuals with diabetes or chronic kidney disease [1]. Hypertension has been identified as one of the risk factors for the emergence of cardiovascular diseases. Past hypertension intervention trials have revealed that the risk treatment of hypertension could reduce the risks of stroke by 42% and a 14% reduction in coronary heart disease [2]. In order to treat hypertension, synthetic drugs have been developed by the pharmaceutical industry. However, such drugs have been reported to produce side effects including insomnia, angioedema, cough and fetal abnormalities [3]. Considering the side effects of synthetic drugs, dietary treatment has gained much attention lately as the alternative antihypertensive approach. Dietary components including prebiotics are seen as better alternatives than drug therapy to treat hypertension considering that prebiotics has a long history of safe use and has been a natural component present in our foods.

Prebiotics are non-digestible food ingredients that can escape digestion under the harsh conditions of the upper gastrointestinal tract and reach the lower gut as substrates for the fermentation by selective indigenous gut microflora. Most of the prebiotics studied are plant derivatives such as fructooligosaccharides (FOS), inulin and fibers. FOS contains 2 to 10 fructose units linked by glycosidic bonds, while inulin is a fructose polymer with β-(2-1) glycosidic linkages with chains of 3 to 60 units. Both FOS and inulin are found abundantly in chicory and artichokes. The major component of chicory root is inulin. Inulin belongs to the fructan family, and occurs naturally as important storage carbohydrates. Other than chicory, fructans are also found present in artichokes, salsify, asparagus and onions [4]. Plant fibers are also found to exert prebiotic properties, health enhancing effects and strongly influence the metabolism of carbohydrates and lipids, which are directly associated with the increased risks of hypertension. Prebiotics from various plant sources have very different chemical structures, physicochemical properties, and physical states. The extraction of prebiotics, especially of plant fiber origin often relies on water solubility or extractability, where plant fibers can be divided into soluble fibers and insoluble fibers [5]. Both soluble and insoluble fibers have been found to exert hypocholesterolemic and hypoglycemic effects that could subsequently lead to an antihypertensive effect.

This paper will discuss some of the antihypertensive mechanisms that have been documents. This paper will also discuss some of the experimental evidence on the antihypertensive properties of plant derived prebiotics, with emphasis on their hypocholesterolemic and hypoglycemia effects. However, controversies have arisen where some studies showed promising results while others exhibited insignificant findings. Thus, this paper will also highlight some of these controversies.

## 2. Antihypertensive Mechanisms of Prebiotics

Various mechanisms have been postulated to explain the ability of prebiotics to reduce the risk of hypertension. One of the possible mechanisms is via the lowering of blood lipid and cholesterol. Previous studies have demonstrated that intensive reduction of cholesterol may be beneficial in the treatment of patients with isolated systolic hypertension [6]. The lipid and cholesterol lowering effects of prebiotics could be attributed to the production of short chain fatty acids (SCFA). It is commonly known that prebiotics resist digestion in the small intestine and reach the colon, where they are fermented by selective colonic microflora to produce SCFA such as acetate, propionate and lactate. The SCFA produced in the large bowel are absorbed in the portal vein, and a major part is metabolized by the liver and subsequently affects various metabolic processes [7]. Indigenous lactic acid bacteria and bifidobacteria often have the ability to ferment these indigestible prebiotics and produce lactate and acetates as the main metabolites, with smaller amounts of propionate and butyrate. It has been reported that propionate could hinder fatty acid and cholesterol synthesis, while lactate produced in the colon plays a significant role in lowering the synthesis of triaclyglycerol fatty acids.

Soluble prebiotics such as pectin, konjac mannan and modified starches are soluble in solutions leading to a thickening and viscous effect. Such physicochemical properties have been found to affect physiological responses such as the lowering of blood cholesterol, and increasing satiety by delaying gastric emptying and a reduced speed of gastric transit in the upper gastrointestinal tract. Levrat-Verny *et al.* [7] evaluated the hypocholesterolemic effects of soluble prebiotics such as guar gum and xanthan gum by using 56 male Wistar rats as a model. The authors found that the administration of 1% w/w of the individual prebiotics for three weeks resulted in increased bile acid flux in feces compared to the control, and a subsequent decrease in plasma and liver cholesterol contents. It was postulated that the soluble prebiotics inhibited intestinal cholesterol and bile absorption by increasing the viscosity of the digesta and the thickness of the unstirred layer in the small intestines. Other than soluble prebiotics, insoluble prebiotics have also been found to exert a hypocholesterolemic effect. They are not soluble, thus could shorten the time of gastric transit and increase the bulk of feces. It has been reported that these insoluble prebiotics could absorb fat and phospholipids in the lower intestines, leading to increased excretion in feces. Cholesterol levels have been reported to be reduced via the binding effect of prebiotics. Gallaher *et al.* [8] proposed that fiber could bind with bile acids and reduce solubilisation of cholesterol leading to a cholesterol lowering effect. The reduction of total cholesterol regulates the receptors of low density lipoprotein (LDL) and thus increases the clearance of LDL cholesterol [9]. This overall cholesterol lowering effect could reduce the stiffness of large arteries and thus could potentially reduce blood pressure [6].

In another study, Lairon *et al.* [10] suggested that the reduction of obesity upon consumption of prebiotics such as fiber could prevent the elevation of blood pressure. Generally, overweighed and obese individuals are at a higher risk of hypertension than those with a healthy body mass index (BMI) [11]. Obesity has also been commonly associated with the overactivity of the sympathetic nervous system where long-term sympatho-activation could raise arterial pressure by causing peripheral vasoconstriction and by increasing renal tubular sodium reabsorption [11]. Therefore, decreasing obesity upon consumption of prebiotics could help to prevent or reduce the risk of hypertension. Past studies involving animals model mainly rats has shown promising evidence that ingestion of inulin-type fructans could regulate body weight via the promotion of endogenous glucagon-like peptide-1 (GLP-1) in the gut [12]. GLP-1 is a key hormone released from enteroendocrine-L cells in response to nutrient ingestion and is the key modulator of food intake by promoting satiety [13]. This consequently reduces the intake of food which leads to a decreased in body weight and BMI. Most of the studies involving the promotion of satiety by fructans via increased production of GLP-1 were performed in animal models and little information is available on human subjects. However, this remains a possible mechanism of fructans in promoting satiety. Piche *et al.* [14] had previously demonstrated that ingestion of 6.6 g of oligofructose three times a day for seven days increased the release GLP-1 in nine subjects. Although the GLP-1 level was not directly associated with satiety in this study, the promotion of GLP-1 in human was a plausible finding of the effects of fructans in human. In addition, it is interesting to note that ingestion of 16g/day of oligofructose for two weeks decreased the total energy intake by 5% and increased satiety following breakfast and dinner in human subjects [15].

Another possible mechanism by which prebiotics could regulate blood pressure includes the attenuation of insulin resistance [16]. Insulin resistance is a metabolic disorder and has been associated with impaired endothelium-dependent vasodilatation which could contribute to increased blood pressure [17]. Therefore, the alteration of insulin sensitivity could potentially protect against the development of this cardiovascular disease. Insoluble prebiotics have been found able to address this issue. Insoluble prebiotics are often extracted from native plant fibers such as cellulose, hemicelluloses, lignin and wheat bran. They have been found to improve postprandial glucose response and decreased secretion of insulin via a lowered glycemic index. The lower circulation of insulin up-regulates the insulin receptors and secondary signalling molecules resulting in increased tissue insulin sensitivity [18]. In addition, other insoluble prebiotics such as high cereal fiber has also been associated with the reduced risk of diabetes, mainly attributed to their metabolism in the colon by indigenous microflora producing SCFA and their effects on hepatic insulin sensitivity [19]. SCFA has been suggested to improve hepatic insulin sensitivity [20]. Cereal fiber also has a low glycemic index and has been studied for their roles in managing diabetes via lowering early postprandial hyperglycemia and decreasing risks of post-absorptive hypoglycemia [21].

Additionally, prebiotics have also been reported to reduce the risk of hypertension by improving the absorption of mineral such as calcium in the gastrointestinal tract [22]. Past studies have shown promising evidence on the correlation of dietary calcium and hypertension. Allender *et al.* [23] conducted a meta analysis of randomized clinical trials on the correlation of dietary calcium and blood pressure and had found that median intake of calcium by 1 g/day could significantly decrease the systolic blood pressure by 1 mm Hg to 2 mm Hg. Diets high in calcium have been found to reduce peripheral vascular resistance and blood pressure leading to a reduced risk of hypertension [24]. However, the beneficial effect of dietary calcium is dependent on its bioavailability and absorption in the intestinal lumen, and the absorption efficiency of calcium in human is low where only 25–30% of the calcium intake is being absorbed by the body [25]. The consumption of prebiotics such as inulin has been reported to enhance the absorption of calcium in human [26]. Prebiotics have been reported to increase the absorption of calcium via binding or sequestering calcium in the upper gastrointestinal tract. Bound or sequestered calcium on prebiotics would then reach the colon where it was being released from the prebiotics matrix and absorbed in the colon [27]. Therefore, the ingestion of prebiotics would increase the absorption of calcium and reduce the risk of hypertension. The absorption of calcium has also been found to increase in the presence of acids in the intestines [28]. Prebiotics are indigestible and thus escape digestion and serve as substrate to the microflora population in the lower intestines. SCFA and H^+^ have been found to exchange for Ca^2+^ in the distal colon regions [29]. This would then increase the concentration of Ca^2+^ which favour passive diffusion and consequently absorbed by the human colon [27].

## 3. *In-Vivo* Evidence of Antihypertensive Effects

Numerous studies have positively indicated that the consumption of plant-derived prebiotics could exert a protective effect against hypertension. Direct association of prebiotics and antihypertension has been established via various *in-vivo* trials (Table 1). However, hypertension is also highly associated with hypercholesterolemia and diabetes, where a lowering in cholesterol levels and improvement of glucose levels has been found to lower blood pressure. Prebiotics have been associated with the promotion of the overall well-being afflicted with blood glucose and blood lipid abnormalities (Table 2), while simultaneously exerting positive effect on blood pressure, thus reducing the risk factors of hypertension and coronary heart diseases.

### 3.1. Hypercholesterolemia

In a study evaluating the influence of prebiotics on cholesterol, Mortensen *et al.* [39] administered a purified diet with 10% of long-chained fructan into male mice for 16 weeks. The control was not fed any prebiotics. The study involved 40 male mice and the results showed that the supplementation of fructan significantly reduced blood cholesterol by 29.7% (*P*<0.001), LDL-cholesterol concentration by 25.9% (*P*<0.01), IDL-cholesterol level by 39.4% (*P*<0.001) and VLDL-cholesterol concentration by 37.3% (*P*<0.05) compared to the control group.

Davidson and Maki [40] conducted a randomized, double-blind, placebo-controlled and crossover design trial for 12 weeks involving 21 healthy volunteers to study the effect of prebiotic on lipid profiles. The authors found that the daily consumption of 18 g/day of inulin-supplemented foods significantly reduced plasma total cholesterol (*P*<0.02) and LDL-cholesterol (*P*<0.005) by 8.7% (± 3.3) and 14.4% (± 4.3), respectively compared to the control. In another study, Rault-Nania *et al.* [41] found that male Wistar rats fed with a diet containing inulin and oligofructose for 4 weeks showed a significant (*P*<0.05) decrease in hepatic triglycerides concentration as compared to those in the control group, whereby the blood pressure of those on inulin and oligofructose were in the range of 138.0 ± 2.2 and 136.9 ± 2.0 mm Hg respectively, while the control showed a blood pressure of 145.8 ± 1.3 mm Hg.

Hypertriglyceridemia is often associated with a moderate hyperglycemia and insulinemia [42], and prebiotics have been found to reduce hepatic triacylglycerol. Daubioul *et al.* [43] administered 10 g of fructan to male mice for eight weeks. The study involved 16 male obese mice and the results showed that the fructan treatment significantly reduced (*P*<0.05) hepatic triacylgylcerol by 48% compared to the control group. In another study, Busserolles *et al.* [44] found that male Wistar-Han rats fed with a diet containing oligofructose for four weeks showed a significant (*P*<0.05) decline of 0.33% in hepatic triacylglycerol as compared to the control. These studies provided the experimental and clinical evidence that the supplementation of prebiotics such as inulin and oligofructose could be used as a mean to control hypertension.

### 3.2. Diabetes

Experimental evidence has also demonstrated that diabetes elevates the risk of hypertension [45]. Dietary intervention is one of the main therapies proposed to diabetics, thus prebiotics have gained increasing attention because of their beneficial effects on lowering blood glucose. Therefore, prebiotics could act as antihypertensive agents upon the exhibition of blood glucose lowering effects. In a study evaluating the improvement of glucose tolerance via the intake of prebiotics, Cani *et al.* [46] administered an oligofructose-supplemented diet [10% (w/w)] to male mice for 14 weeks. The study used 32 male mice and the results showed that mice in the group consuming diet supplemented with oligofructose had improved glucose tolerance as compared to those in the control group. Mice on the oligofructose-supplemented diet showed normal fasting plasma insulin levels and restored glucose-induced insulin secretion while the control did not. In another study, Frias and Sgarbieri [47] found that male Wistar rats (n=120) fed with diet containing 20% (w/w) of guar gum for 60 days showed a significantly (*P*<0.05) reduced serum glucose. Similarly, Kok *et al.* [48] studied the effects of oligofructose-enriched diet on glucose metabolism using rats as a model. The study involved 22 male Wistar rats that were fed a nonpurified diet and were randomly divided into two groups: those receiving nonpurified diet with 10% oligofructose (treatment group), or without oligofructose (control group) for 30 days. Throughout the 30-day treatment, the treatment group showed a significantly (*P*<0.05) reduced blood glucose by 26.0% as compared to those of the control group which only showed a reduction of 9.7%, which was insignificant statistically. The finding indicated that diet supplemented with oligofructose was effective in reducing blood glucose and indirectly could alleviate the risks of hypertension.

Similarly, Suzuki and Hara [49] studied the effects of guar gum hydrolysate-enriched diet on glucose tolerance using rats as a model. The study used 31 male Sprague-Dawley rats that were fed a diet containing guar gum hydrolysate (75 g/kg) for 30 days. The treatment contributed to a significant (*P*<0.05) improvement of glucose intolerance as compared to the control. Giacco *et al.* [50] evaluated the effect of short-chain-fructooligosaccharides on glucose tolerance in 30 volunteers. The randomized, double-blind, placebo-controlled and crossover trial for two months showed that daily consumption of 10 g/day of short-chain-fructooligosaccharides contributed to a significant (*P*<0.02) reduction of postprandial insulin response as compared to those in the placebo group which did not show any significant differences.

Various studies have highlighted the beneficial effects of prebiotics on physiological conditions such as lipid and glucose profiles that are directly associated with hypertension. Hence, there is a strong basis for continuous evaluation on prebiotics specifically aimed at utilizing longer and larger *in-vivo* trials.

## 4. Controversial Studies

Epidemiological studies have found that hypertensive patients frequently have a high level of serum cholesterol. Blood pressure and cholesterol are closely related and many researches have demonstrated that the lowering of cholesterol contributes to antihypertension effects. Thus, an increase in both serum cholesterol and triglycerides had been reported to elevate blood pressure values [51]. According to Boyd *et al.* [52] and Ferrara *et al.* [51], blood pressure elevated with a higher prevalence of low HDL-cholesterol and high LDL-cholesterol levels while mildly increased serum cholesterol levels are able to influence hypertension. Although increasing uptake of prebiotics have been reported to reduce plasma lipid levels, controversies are raised where a number of studies have reported otherwise.

According to Luo *et al.* [53], blood lipids in twelve healthy volunteers were not affected by the consumption of nondigestible oligosaccharides (NDO) such as short-chain fructooligosaccharides (FOS). The consumption of FOS enriched cookies (20 g/day) in a double-blind randomized crossover design over 4 weeks showed that serum triacylglycerol, total- and HDL cholesterol, apoliprotein A-I and B and lipoprotein concentrations remained relatively constant and did not differ compared to the control. In another randomized, double-blind and diet-controlled study involving twelve healthy men, Van Dokkum *et al.* [54] showed that the cholesterol lowering effect of NDO was insignificant. The incorporation of inulin, FOS and galacto-oligosaccharides (GOS) in food products in quantities up to 15 g/day did not exhibit a reduction in blood lipid profiles. Although factors such as healthy subjects and low concentrations of initial serum cholesterol could affect the outcomes of these studies, the results showed that NDO may not possess antihypertensive effects.

In another study, Pedersen *et al.* [55] reported that the daily intake of 14 g of inulin added to a low fat spread in the diet of sixty-four healthy young females in a randomized double-blind crossover study involving two periods of four weeks did not affect the concentrations of triacylglycerols, HDL and LDL-cholesterol. There was insignificant difference in blood lipids between the placebo and treatment group. Ellegard *et al.* [56] also showed similar findings. In a double-blind crossover study, the consumption of 16–17 g/d of inulin and FOS in a controlled diet by ten subjects with conventional ileostomy did not show significant effects on absorption and excretion of cholesterol. In another study by Williams [57], fifty-eight middle-aged hypercholesterolemic subjects consuming 10 g/day of inulin showed insignificant changes in total, LDL or HDL-cholesterol or apolipoproteins B and A over eight weeks.

Jenkins *et al.* [58] used a crossover design involving eight healthy subjects and showed that daily ingestion of 18–25 g/day of lactulose in dissolved water over two weeks not only did not reduce lipid concentrations but raised the fasting serum total- and LDL-cholesterol and apoliprotein B concentrations. The authors reported that the failure of lactulose to lower serum lipids may related to inadequate generation of propionate and predominant of acetate short chain fatty acids (SCFA) because six of the eight subjects showed higher serum acetate concentrations. In another animal study involving rats as a model also showed a significant rise in liver cholesterol concentrations after four weeks of dietary supplementation with lactulose [59]. Fermentable dietary fibers such as prebiotics escape digestion in small intestine and are fermented in the cecum and colon, producing SCFA mainly acetate, propionate and butyrate [60]. According to Beynen *et al.* [61], acetate is a precursor of cholesterol synthesis and it has been shown to enhance lipogenesis in rat hepatocytes.

The concordance of hypertension and diabetes is increased in the population and hypertension is disproportionately higher in diabetics [62]. High blood glucose levels in hyperglycemia patients are more likely to elevate blood pressure [63]. Although prebiotics have been widely reported to improve blood glucose profiles, some studies have also suggested otherwise. Alles *et al.* [64] previously used a randomized, single-blind, placebo-controlled and crossover design involving twenty patients with type 2 diabetes. The authors reported that the supplementation of FOS (15 g/d) for 20 days did not significantly affect blood glucose concentrations in patients with type 2 diabetes. Luo *et al.* [65] also showed similar findings in a trial involving ten type 2 diabetic volunteers. These subjects received 20 g/d FOS for four weeks in a double-blind crossover design. The authors found that FOS did not modify plasma glucose and insulin concentrations or basal hepatic glucose production. The authors attributed the absence of glucose lowering effect of FOS to the relatively small doses used in the subjects. In a randomized, single-blind and crossover study involving eighteen mildly hyperlipidemic subjects, Keogh *et al.* [66] demonstrated that the administration of β-glucan enriched barley for two period of four weeks did not significantly change blood glucose levels. In another study, McIntosh *et al.* [67] evaluated the effect of blood glucose upon the consumption of dietary fiber in a crossover trial involving twenty-one mildly hypercholesterolemic subjects. The authors found that the daily intake of 21–38 g of barley foods containing β-glucan and wheat containing cellulose and hemicelluloses fiber for four weeks did not alter the concentrations of blood glucose.

Future studies aimed at investigating the effects of prebiotics on serum cholesterol or blood glucose concentration should consider the choice of subjects and the length of the supplementation period. Past studies investigating on the effects of NDO such as inulin and FOS in humans on hypertension remains relatively controversial. However, considering the vast experimental evidence on their positive roles, the potential of prebiotics as an antihypertensive agents warrant further investigations.

## 5. Conclusions

Results from recent studies support the antihypertensive potential of plant-based prebiotics, and shown that they could exert such a beneficial effect via various mechanisms. Although controversial findings are raised, positive outcomes from both *in-vitro* experiments and *in-vivo* trials have exhibited the need for further evaluation of the antihypertensive properties of plant-based prebiotics.

## Figures and Tables

**Table 1.** Antihypertensive effects of plant derived prebiotics.

**Intervention**	**Experimental design**	**Subjects**	**Dose**	**Effects**	**Ref**
Soluble fiber extracted from oat bran	Randomized, double-blind, placebo-controlled	n=110; 30 to 65 years; not on hypertension treatment; SBP of 125–159 mmHg and DBP of < 95 mmHg	8 g/d of fiber for12 weeks	A reduction in SBP of 2.0 mmHg and DBP of 1.0 mmHg	[30]
Diet containing soy protein isolate and supplementation of fiber extracted from psyllium	Randomized, double-blind, parallel	n=36; nonsmoking men or women > 20 years old; on antihypertensive drug therapy for > 6 months; SBP of 130– 160 mmHg	12 g fiber/d for 8 weeks	A reduction in SBP of 5.9 mmHg	[31]
Dietary fiber in the form of pill supplementation	Randomized, double-blind, parallel, placebo-controlled	n=63; 18–70 yrs old; hypertensive with a minimum DBP of > 90 mmHg	7 g/d of dietary fiber for 12 weeks	A reduction in DBP of 5 mmHg	[32]
Beta-glucan from whole oats cereals	Randomized, parallel, pilot trial	n=18; 27–59 years old; healthy, untreated hypertensives with SBP of 130–160 mmHg and DBP of 85–100 mmHg	5.52 g/d of beta- glucan for 6 weeks	A reduction in SBP of 7.5 mmHg and DBP of 5.5 mmHg	[33]
Bread substituted with lupin kernel flour	Randomized, parallel	n=74; 20–70 years old; overweight and obese men and women with BMI of 25–35; SBP<150 mmHg and DBP<95 mmHg	4 x 40g of bread/d for 16 weeks; Bread contained 9.5% w/w of fiber	A reduction in SBP of 3.0 mmHg	[34]

**Table 2.** Effects of dietary fiber on blood glucose and lipid profiles.

**Intervention**	**Fiber**	**Dose; duration of the study**	**Experimental design**	**Animals/Subjects**	**Effects**	**Ref**
Blood Glucose	Alginate fiber	5.0-g sodium alginate supplement (algaeisolate, 75% soluble fiber); for two days	Randomized, placebo-controlled	Seven men with type 2 diabetes; mean age of 53 years	Significantly reduced the postprandial rise in blood glucose (*P*<0.05) and in serum insulin (*P*<0.02) by 31% and 42%, respectively	[35]
	Soy hulls	26 g of soy hulls which incorporated into 7 slices of bread daily; for 4 weeks	Randomized, double-blind, placebo- controlled	Ten subjects (5 male and 5 female) with type 2 diabetes; mean age of 65 ± 5.9 years	Significantly improved the glucose score (*P*<0.05) and the total area under the glucose curve (*P*<0.05) by 6.7% and 7.1%, respectively	[36]
Lipid Profile	Pectin	75 g citrus pectin daily; for four weeks	Randomized, placebo-controlled	Six male adult hypercholesterolemic minipigs	67.1% decrease in VLDL- cholesterol (*P*<0.05); 41.1% decrease in LDL- cholesterol (*P*<0.05); 49.4% decrease in total serum cholesterol (*P*<0.05)	[37]
	Fiber (*Plantagoovata* husk)	10.5 g *Plantago ovata* husk daily; for eight weeks	Randomized, crossover, placebo-controlled, single-blind	Twenty-eight men with myocardial infarction or stable angina	6.7% decrease in plasma triacylglycerol (*P*<0.02), 6.7% increase in HDL-cholesterol concentrations (*P*<0.006); 10.6% decrease in the total cholesterol/HDL ratio (*P*<0.002); 14.2% decrease in LDL/HDL ratio (*P*<0.003)	[38]
